# The WRKY transcription factor family and senescence in switchgrass

**DOI:** 10.1186/s12864-015-2057-4

**Published:** 2015-11-09

**Authors:** Charles I. Rinerson, Erin D. Scully, Nathan A. Palmer, Teresa Donze-Reiner, Roel C. Rabara, Prateek Tripathi, Qingxi J Shen, Scott E. Sattler, Jai S. Rohila, Gautam Sarath, Paul J. Rushton

**Affiliations:** Texas A&M AgriLife Research and Extension Center, Dallas, TX 75252 USA; Grain, Forage and Bioenergy Research Unit USDA-ARS UNL, Lincoln, NE 68583-0937 USA; Department of Biology, West Chester University of Pennsylvania, West Chester, PA 19382 USA; Molecular and Computational Biology Section, Dana & David Dornsife College of Letters, Arts and Sciences, University of Southern California, Los Angeles, CA USA; School of Life Sciences, University of Nevada Las Vegas, Las Vegas, NV 89154 USA; Department of Biology and Microbiology, South Dakota State University, Brookings, SD 57007 USA; Current address: 22nd Century Group Inc., Clarence, 14031 New York

## Abstract

**Background:**

Early aerial senescence in switchgrass (*Panicum virgatum*) can significantly limit biomass yields. WRKY transcription factors that can regulate senescence could be used to reprogram senescence and enhance biomass yields.

**Methods:**

All potential WRKY genes present in the version 1.0 of the switchgrass genome were identified and curated using manual and bioinformatic methods. Expression profiles of WRKY genes in switchgrass flag leaf RNA-Seq datasets were analyzed using clustering and network analyses tools to identify both WRKY and WRKY-associated gene co-expression networks during leaf development and senescence onset.

**Results:**

We identified 240 switchgrass WRKY genes including members of the RW5 and RW6 families of resistance proteins. Weighted gene co-expression network analysis of the flag leaf transcriptomes across development readily separated clusters of co-expressed genes into thirteen modules. A visualization highlighted separation of modules associated with the early and senescence-onset phases of flag leaf growth. The senescence-associated module contained 3000 genes including 23 WRKYs. Putative promoter regions of senescence-associated WRKY genes contained several *cis*-element-like sequences suggestive of responsiveness to both senescence and stress signaling pathways. A phylogenetic comparison of senescence-associated WRKY genes from switchgrass flag leaf with senescence-associated WRKY genes from other plants revealed notable hotspots in Group I, IIb, and IIe of the phylogenetic tree.

**Conclusions:**

We have identified and named 240 WRKY genes in the switchgrass genome. Twenty three of these genes show elevated mRNA levels during the onset of flag leaf senescence. Eleven of the WRKY genes were found in hotspots of related senescence-associated genes from multiple species and thus represent promising targets for future switchgrass genetic improvement. Overall, individual WRKY gene expression profiles could be readily linked to developmental stages of flag leaves.

**Electronic supplementary material:**

The online version of this article (doi:10.1186/s12864-015-2057-4) contains supplementary material, which is available to authorized users.

## Background

Switchgrass (*Panicum virgatum*) is a temperate, warm-season perennial that is being developed as a cellulosic biofuel crop [1, 2]. Tetraploid switchgrass populations and cultivars have higher yields as compared to octaploid populations [3]. Thus, most current breeding efforts are focused on improving biomass yields and quality in tetraploid lines [4, 5]. Tetraploid populations can occur as upland and lowland ecotypes, with the lowland plants significantly out-yielding the upland lines across several locations [5]. However, the latitudinal adaptation of these different ecotypes presents challenges, since most of southerly-adapted, high-yielding, lowland germplasm suffers from significant winter-kill at more northern sites of the USA [6]. Some crosses of upland x lowland plants show heterosis for yields [7], and this approach appears to hold promise in the continuing development of switchgrass as a biomass crop [8]. Nevertheless, extending the period of carbon assimilation by delaying aerial senescence could be a means to significantly improve yields, as long as other plant attributes, such as dormancy onset and nutrient remobilization are not impaired [6].

Senescence is a genetically programmed trait that can potentially be reprogrammed by several molecular breeding strategies such as marker-assisted selection. To develop switchgrass cultivars with delayed senescence, it is critical to determine the key molecular events that occur during senescence to identify the regulators that trigger this process. Senescence is the final stage of plant development and is tightly controlled to increase the fitness of the whole plant [9]. Transcriptome analysis of *Arabidopsis thaliana* (*A. thaliana*; At) leaf senescence suggests that several families of transcription factors play major roles in the cellular reprogramming associated with senescence. The major transcription factor families associated with *A. thaliana* leaf senescence are NACs, WRKYs, C2H2 zinc finger proteins, AP2/ERFs, MYBs, homeobox proteins, bZIPs, bHLHs, and C3H zinc finger proteins. WRKY TFs were the second largest TF family to be induced during senescence in this study [10].

WRKY transcription factors are key regulators of many plant processes, including responses to biotic and abiotic stresses, wounding, senescence, seed dormancy, and seed germination [11]. They are components of intracellualar signaling webs, for example many are phosphorylated by MAP kinase cascades [12]. The defining feature of WRKY transcription factors is their DNA binding domain referred to as the WRKY domain, which is named after the almost invariant WRKY amino acid sequence within the N-terminal region [13]. The WRKY domain is about 60 residues in length and also possesses a Cx_4–5_Cx_22–23_HxH or Cx_7_Cx_23_HxC zinc-finger structure at the C-terminus [11]. Structural determination of the WRKY domain bound to its W box *cis*-acting element revealed that part of a four-stranded β-sheet enters the major groove of DNA almost perpendicular to the DNA helical axis in a β-wedge. Amino acids in the conserved WRKYGQK signature motif contact the W box DNA bases [14].

Functional genomic studies of individual WRKY transcription factors have provided clear evidence that specific WRKY proteins are regulators of senescence, although some of these transcription factors play multiple roles *in planta* [15–17]. The first evidence supporting a role of WRKY transcription factors in the senescence process came from studies of *A.thaliana AtWRKY6* [18, 19]. One target gene for *AtWRKY6* is *FLG22-induced receptor-like kinase 1* (*FRK1* previously called *SIRK*) whose expression is strongly induced during leaf senescence. Senescing leaves of *wrky6* knockout mutants showed a drastic reduction in *FRK1* transcript levels and green leaves of *WRKY6* overexpression lines showed clearly elevated *FRK1* transcript levels. In *A.thaliana*, *AtWRKY54* and *AtWRKY70* appear to have cooperative and partly redundant functions in senescence, as revealed by single and double mutant studies [20]. *AtWRKY54* and *AtWRKY70* are both negative regulators of senescence and interact independently with *AtWRKY30* which is expressed during developmental leaf senescence [20]. Another member of the WRKY family in *A.thaliana*, *AtWRKY53*, acts as a convergence node between senescence and pathogen responses [21]. The AtWRKY53-interacting protein UPL5 is a HECT domain E3 ubiquitin ligase that regulates leaf senescence in *A.thaliana* through degradation of AtWRKY53, demonstrating that targeted breakdown of AtWRKY53 is a feature of senescence in *A.thaliana*. Recently it has been shown that AtWRKY18 represses *AtWRKY53* activity and acts as a positive regulator of senescence [22]. *AtWRKY22* has also been implicated in regulating dark-induced leaf senescence and appears to share cross-regulation with *AtWRKY6, AtWRKY53,* and *AtWRKY70* [23]. Other WRKY transcription factors that have been implicated in regulating senescence include rice *OsWRKY42* [24], *OsWRKY80* [25], and *OsWRKY23* [26]. Taken together, studies of both specific WRKY transcription factors and the WRKY family as a whole demonstrate that WRKY proteins play important roles in regulating the process of senescence.

In this study, the members of the WRKY gene family that are present in Version 1.1 of the genome sequence of switchgrass (www.phytozome.org) were identified. The names and genomic locations were enumerated for 191 full-length WRKY genes, together with 49 partial WRKY genes where complete sequence of the gene was lacking. Some of these 49 genes are likely pseudogenes due to the presence of nonsense mutations within the ORF and missing portions of the WRKY domain. Switchgrass also contains a Group RW5 R protein-WRKY gene, consisting of a domain structure of B3-LRR-NB ARC-LRR-WRKY. The presence of a B3 DNA-binding domain together with a WRKY domain and domains from intracellular resistance proteins suggest that this R protein has at least two different DNA-binding domains with different *cis*-acting element specificities that could be responsive to both biotic and abiotic stresses. We also show that the switchgrass genome contains a second R protein-WRKY gene. The PviWRKY174 protein is a Group RW6 protein orthologous to sorghum SbRWRKY2 and SbRWRKY3. Existing RNA-Seq datasets [27] from flag leaves obtained from field grown switchgrass plants at distinct stages of development were used to understand the relationships between WRKY gene expression and leaf developmental state. Using a range of bioinformatic analyses, distinct modules of co-expressed genes were found to be associated with specific flag leaf developmental stages. A co-expressed cluster of 3000 genes containing 23 WRKYs were specifically associated with the onset of senescence.

## Methods

### Sequence data sets

The sequences of the complete WRKY gene family from switchgrass were taken from v1.1 genome sequence at Phytozome (http://www.phytozome.org/) [28]. These sequence data were produced by the US Department of Energy Joint Genome Institute. Senescence associated WRKY genes from other species were obtained from the Leaf Senescence Database (http://www.eplantsenescence.org/) by performing a text search with the term “WRKY”.

### Identification and manual curation of the switchgrass WRKY transcription factor family

To identify the WRKY family in switchgrass, a modification of the TOBFAC pipeline was used [29]. Tblastn searches were performed against the JGI release v1.1 of the switchgrass genome sequence using a representative WRKY domain from each of the flowering plant subfamilies of WRKY transcription factors (I, IIa, IIb, IIc, IId, IIe, and III) [29]. The e-value was set to 10 to ensure that all potential WRKY domain-encoding sequences, however diverse or fragmentary, were discovered. All hits were pooled into a single data set and duplicate sequences were then removed. For each positive genomic sequence, about 20 kb of genomic sequence around the WRKY domain-encoding region (if available) was used in the gene prediction program FGENESH (http://www.softberry.com/) with the monocot plant setting and the resultant amino acid prediction compared to the gene model (if present). Positive genomic sequences were also analyzed by Hidden Markov Model analyses using the protein sequence vs profile-HMM 624 database tool at Janelia.org (http://hmmer.janelia.org). For this analysis the default settings of the program were used to search the Pfam, Gene3D, and Superfamily databases. The R protein-WRKY genes were further investigated by blastp, PSI-BLAST and tblastn searches at NCBI (http://www.ncbi.nlm.nih.gov/) [30, 31].

### Phylogenetic analyses

The amino acid sequences of the WRKY domains or the complete amino acid sequences of the R protein-WRKYs [32] were used for phylogenetic analyses. Alignments were constructed using MUSCLE [33] with the following parameters; Gap Penalties: Gap open −2.9, Gap Extended 0, Hydrophobicity multiplier 1.2 Memory/Iterations: Max Memory in MB 4095, Max Iterations 8; Clustering Method Iteration 1, 2 (UPGMB), Clustering Method (Other Iterations (UPGMB), Min. Diag. Length (Lambda) 24. For the Neighbor Joining tree [34], the percentage of replicate trees in which the associated taxa clustered together in the bootstrap test (1000 replicates) were determined [35]. The evolutionary distances were computed using the Poisson correction method [36] and are in the units of the number of amino acid substitutions per site. All ambiguous positions were removed for each sequence pair. Evolutionary analyses were conducted in MEGA6 [37]. All positions containing alignment gaps and missing data were eliminated in pairwise sequence.

### Switchgrass Flag Leaf RNA-Seq Data

Previously published [27] RNA-Seq data from switchgrass was used to analyze WRKY expression (SRA Accession SRX481052). Briefly, flag leaves from field grown cv Summer plants were collected in 2012 at five time points: heading (July 3), anthesis (July 27), seed set (August 16), mature seed (August 31), and senescence onset (September 19). At each time point, three pools of 10 flag leaves each were collected from randomly selected plants. RNA was isolated from all samples and 100 bp single-end sequencing was performed using an Illumina HiSeq2000 instrument with five samples per lane, yielding an average of 45 million reads per sample.

### Mapping and differential gene expression analysis

HiSeq2000 100-bp reads were mapped to version 1.1 of the switchgrass genome (www.phytozome.org). Tophat2 (version 2.0.11) [38] was used with default parameters for mapping and reads with multiple alignments were discarded prior to counting gene expression, whereby only uniquely mapped reads were used for all subsequent analyses. Expression values were calculated using the featureCounts function in the Subread (version 1.4.4) analysis program [39], along with the version 1.1 gene annotation file which was modified to include WRKY genes identified as already described. Differentially expressed genes (FDR < 0.05) across the time series were identified using the likelihood ratio test in DESeq2 (version 1.6.3) [40, 41] in R [42].

### NMDS and Hierarchical Clustering Analysis

Raw read counts were normalized and subjected to the variance stabilization transformation from DESeq2 to enable comparisons of expression levels between WRKY genes and to correct for differences in gene lengths. Standardized counts of WRKY genes were analyzed via NMDS using the ‘metaMDS’ function from the vegan package [43] in R (version 3.1.1 for Linux) to determine if WRKY expression profiles changed over the course of development time in coordinated manner and to ascertain whether flag leaves collected at the same developmental time points displayed similar WRKY expression patterns.

To identify clusters of WRKY genes that were activated during each flag leaf developmental stage, variance stabilized counts of differentially expressed WRKY genes that were obtained above were also subjected to agglomerative hierarchical analysis as follows: Variance-stabilized read counts obtained in the previous section were log transformed and Z-scores were computed. A compositional Euclidean dissimilarity matrix was computed and clustering analysis was performed using Ward’s method on z-scores derived from the average of the replicates collected at each time point in JMP® Version 9.0 (SAS Institute Inc, Cary, NC, 1989–2007).

### Co-expression Network Analysis

Weighted Gene Co-expression Network Analysis (WGCNA, version 1.43), an R package, was used to identify groups of genes having similar expression patterns across the flag leaf time series [44, 45]. Differentially expressed genes with a log_2_ fold change of 1.5 or greater were used for network analysis (19,049 total genes). A soft threshold (β) value of 12 was used in the transformation of the adjacency matrix in order to meet the scale-free topology criteria. Co-expression modules were created with the blockwiseModules function using the following parameters: TOMType=”unsigned”, maxBlockSize = 20000, mergeCutHeight = 0.4, minModuleSize = 15. The expression pattern of the resulting modules is represented by the module eigengene (ME), which corresponds to the first principal component of a given module.

### Module Visualization

Cytoscape (version 3.2.0) [46] was used to visualize co-expression networks identified using WGCNA. The topological overlap measure (TOM) calculated by WGCNA was used as a measure of co-expression for pairs of genes. Prior to visualization, the overall network size was reduced in two ways. First, all gene pairs were filtered by requiring one of the two genes to be a transcription factor. Putative switchgrass transcription factors were identified by PFAM [47] annotations and following the family assignment rules detailed in the Plant Transcription Factor Database v3 [48]. A total of 901 putative transcription factors (including WRKYs) were identified in the flag leaf time series differentially expressed gene list. Second, the top 2.5 % of the TOM values for each gene pair were retained. The final network contained 13,405 nodes (genes) connected by 428,378 edges (TOM values). The network was drawn in Cytoscape using the AllegroLayout plugin with an edge-weighted Allegro Fruchterman-Reingold algorithm.

### Promoter Analysis of Genes of Interest

To identify potential *cis*-regulatory elements in WRKY genes induced during senescence, the promoter regions of the senescence-related WRKY genes, defined as 1000 bp upstream of the start codon, were scanned for putative regulatory motifs using Place Web Signal Scan (https://sogo.dna.affrc.go.jp/cgi-bin/sogo.cgi?lang=en&pj=640&action=page&page=newplace) [49, 50]. While promoter regions could not be identified for several of the WRKY genes, which were directly adjacent to scaffold boundaries or were located on unplaced contigs, we were able to extract full 1000 bp promoter regions for 17 of the 23 WRKY genes that were activated during flag leaf senescence.

### Availability of supporting data

The data used in this manuscript are available as part of the short-reads archive depository within the NCBI at http://www.ncbi.nlm.nih.gov/sra/SRX481052/

## Results

### The WRKY transcription factor family in switchgrass

To identify all members of the WRKY gene family in the switchgrass (v1.1) genome sequence, the same modified version of the TOBFAC pipeline used previously to identify WRKY genes in *Brachypodium distachyon* [29] was employed. Briefly, tblastn searches [30] were performed against the *Panicum virgatum* v1.1 genome sequence using a representative WRKY domain from each of the subfamilies of WRKY transcription factors found in flowering plants (I, IIa, IIb, IIc, IId, IIe, and III). These multiple searches employed a cut off e-value of 10 in order to identify all possible WRKY domain encoding sequences even if these sequences were incomplete. All of the positive sequences were combined into a single dataset and redundant sequences were removed. Finally, every sequence was manually curated to ensure that each sequence contained at least part of a WRKY domain and could therefore be regarded as a switchgrass WRKY gene. This pipeline enabled us to produce a data set of switchgrass WRKY genes that is more complete than the predicted gene models in the v1.1 genome sequence. For each positive genomic sequence, about 20 kb of genomic sequence around the WRKY domain-encoding region (if available) was used in the gene prediction program FGENESH with the monocot plant setting and the resultant amino acid prediction was compared to the gene model (if present in phytozome). Positive genomic sequences were also analyzed by Hidden Markov Model analyses using the protein sequence vs profile-HMM 624 database tool at Janelia.org (http://hmmer.janelia.org). Using this pipeline we identified 191 full length WRKY genes and named them *PviWRKY1*-*PviWRKY191* (Additional file 1: Table S1). We gave the genes PviWRKY names to avoid confusion with *Phaseolus vulgaris*, the common bean, whose genome sequence is publically available. In addition, we found 49 WRKY domain-containing sequences that did not encode a full length gene. Inspection of these incomplete sequences revealed that many were located on short contigs and were therefore lacking the complete genomic sequences. Several others were present on chromosomal sequences but coding regions were interrupted by regions of “Ns” and therefore have missing portions of the gene. This situation is not surprising as version 1.1 of the switchgrass genome contains 636.1 Mb of sequence localized to chromosomes with an additional 593.5 Mb which is not localized. The *Panicum virgatum* Genome Sequencing Project expects that there will be significant future movement of genes as they integrate direct sequence from the clone based genome improvement project (http://phytozome.jgi.doe.gov) into future assemblies. The total number of WRKY sequences identified in the *P.virgatum* genome assembly was 240. Of the 191 complete WRKY genes, nine were completely lacking a gene model in phytozome and three had gene models that incorrectly predicted the WRKY domain. The forty nine incomplete WRKY genes were named *PartialWRKY1*-*PartialWRKY49* and will be added to the list of complete genes or pseudogenes when additional sequence data become available. Of these 49 sequences, 27 had a partial gene model, suggesting that they could be functional. RNA-Seq data collected from switchgrass flag leaves detected transcripts from 37 partial WRKYs and 169 full-length WRKYs indicating a final number of at least 206 expressed WRKY genes in switchgrass leaves (Additional file 1: Table S1).

### Resistance protein-WRKY genes in the switchgrass genome

**Fig. 1:**
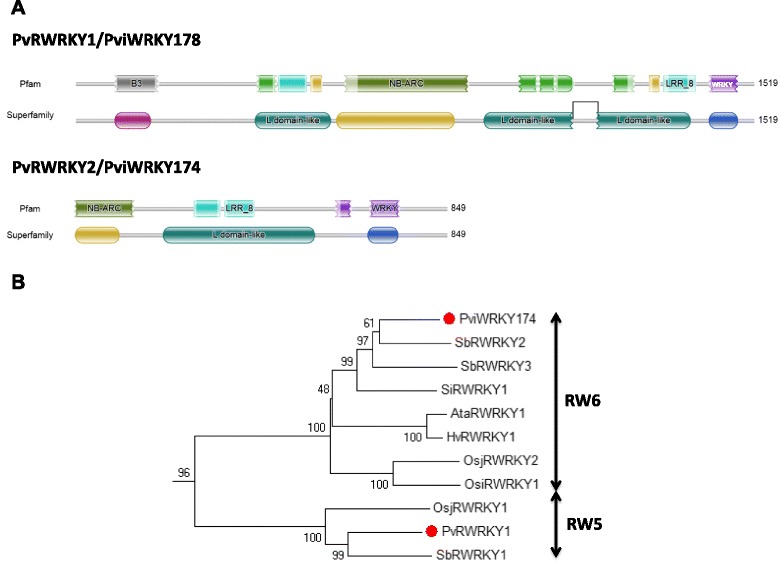
Resistance protein – WRKY (RW) genes in switchgrass. **a** Domain structures of PvRWRKY1/PviWRKY178 and PvRWRKY2/PviWRKY174. Hidden Markov Model analyses were performed with the complete amino acid sequences using the protein sequence vs profile-HMM database tool at Janelia.org (http://hmmer.janelia.org) and searching the Pfam and Superfamily databases. **b** Neighbor Joining phylogenetic tree derived from a MUSCLE alignment of full length R protein-WRKYs from Groups RW5 and RW6. Numbers indicate bootstrap values from 1000 replicates. Red dots denote switchgrass proteins

One key feature of the WRKY gene family in many, but not all, species of flowering plants is the existence of chimeric proteins comprising domains typical for both resistance (R) proteins and WRKY transcription factors. An atlas of R protein-WRKY genes has been assembled and the proteins classified into eight groups [32]. These groups of R protein-WRKYs have been named RW1-8 and new groups can be expected to be discovered as more plant genome sequences become available. The switchgrass WRKY transcription factor *PviWRKY178* encodes a Group RW5 protein of the domain structure B3-LRR-NB ARC-LRR-WRKY (Fig. 1) that we have also previously called *PvRWRKY1* [32]. This gene was expressed in flag leaves (see below). This gene is a distinct type of R protein-WRKY because it contains two different types of DNA-binding domain (a WRKY domain and a B3 domain). The B3 domain has previously been identified in three major classes of transcription factors, ABI3/VP1-like factors, the RAV-like family, and auxin response factors (ARFs) [51] but Group RW5 proteins are the first reported proteins that combine B3 domains and WRKY domains. The B3 domain proteins play roles in the responses to abscisic acid and auxin [52, 53] but the role of *PviWRKY178/PvRWRKY1* is currently unknown.

Detailed searches also revealed that *PviWRKY174* also encodes an R protein-WRKY and that it had previously been overlooked [32] as it is located on a short contig of only 6.13 kb (Additional file 1: Table S1). Nevertheless, HMMER analysis suggests that the complete coding sequence may be present and phylogenetic analyses demonstrate that the PviWRKY174 protein is a Group RW6 protein similar to proteins encoded by sorghum *SbRWRKY2* and *SbRWRKY3* (Fig. 1). Therefore the switchgrass genome contains two expressed RW genes, one belonging to Group RW5 and one to group RW6. Both sorghum (*Sorghum bicolor*) and rice (*Oryza sativa*) also contain RW genes from these two groups and this may be a feature of some, but not all monocot genomes. For example, the *Brachypodium distachyon* genome appears to lack RW genes entirely.

This comprehensive analysis of the WRKY family of genes and proteins in switchgrass permitted a deeper enquiry of existing transcriptomic datasets to quantitate specific WRKY gene expression during flag leaf development in field grown switchgrass plants. Our intent was to (a) describe the overall patterns of WRKY gene expression at specific stages of leaf and plant development; and (b) to analyze gene networks associated with leaf senescence with the goal of discovering WRKYs that could activate flag leaf senescence.

The dataset used in the current study was obtained from flag leaves [27] collected from plants at heading (7/3/12); anthesis (7/27/12); early seed set (8/16/12); seeds at hard seed set (8/31/12); and at physiological maturity when flag leaf senescence was visually obvious (9/19/12). Global aspects of these data sets have been described in Palmer et al. (2014). A total of 110 WRKYs were differentially expressed across all harvest dates.

### Non-metric multidimensional scaling (NMDS) analysis of WRKY gene expression

**Fig. 2:**
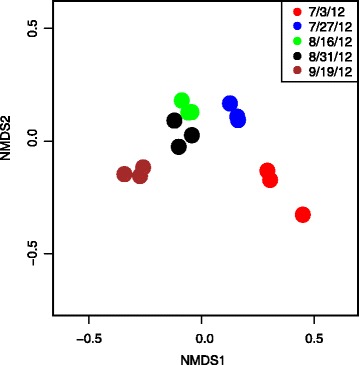
Non-metric multidimensional scaling (NMDS) analysis of WRKY expression patterns in switchgrass flag leaves collected across five different developmental states. The three biological replicates at each harvest date are shown in the same color

To determine whether the WRKY expression profiles were correlated with flag leaf development, NMDS ordination based on a Bray-Curtis dissimilarity matrix of the standardized expression levels of the differentially expressed (DE) WRKY genes in switchgrass flag leaves was performed. This analysis (Fig. 2) demonstrated that WRKY gene expression levels in flag leaves collected at each developmental stage (harvest date) were distinct and that individual replicates within each time point were quite similar to each other, indicating that the expression levels of WRKY genes were highly consistent in flag leaves at the same stages of development. Furthermore, the DE-WRKY expression profiles of young expanding flag leaves (red circles, Fig. 2) were separated from the DE-WRKY expression profiles of flag leaves collected at other harvest times. DE-WRKY expression patterns were also more similar in flag leaves as they transitioned to source leaves (blue circles, Fig. 2) compared to other harvest dates when flag leaves were expected to be fully functional in terms of carbon assimilation and transport (green and black circles, Fig. 2). The first and last harvest dates were most differentiated by the NMDS axis 1, when flag leaves were elongating or had started to senesce respectively. These results indicated that the WRKY expression profile changed in a coordinated manner over the course of flag leaf development and WRKYs contributing to senescence could be identified.

### Hierarchical Clustering Analysis

**Fig. 3:**
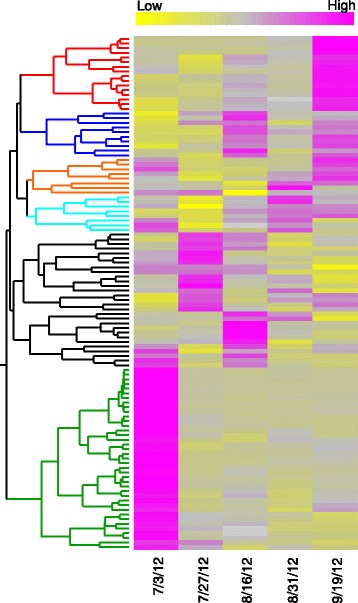
Hierarchical clustering analysis of 110 differentially expressed WRKY genes. The Z-score heatmap was derived from the average of the replicates collected at each time point (displayed in Fig. 2). Yellow is low expression; Magenta is high expression

Because the NMDS analysis indicated that activation of specific WRKY genes may stimulate senescence in flag leaves, a hierarchical clustering to identify groups of senescence-related WRKY genes was conducted (Fig. 3). Two distinct clusters of WRKY genes whose expression levels were elevated during senescence were observed (Additional file 1: Table S1). The first cluster (red) included 16 genes whose expression levels were elevated on the 9/19/12 collection date compared to other time points, indicating that their induction is likely linked to senescence in flag leaves. The second cluster (blue) included 10 genes (Additional file 2: Table S2) whose expression levels were highest on 8/16/12 and 9/19/12 time points. A third cluster (orange) included 8 WKRY genes whose expression levels were elevated at both the 7/13/12 and 9/19/12 time points, although their expression levels were significantly increased on 9/19/12 relative to 7/13/12, potentially linking them to leaf senescence. A fourth cluster (cyan) included 8 WRKY genes with variable patterns of expression levels. Of significance are three WRKY genes whose expression levels were elevated on 8/31/12 and 9/19/12 (Fig. 3). Notably, a large cluster containing 39 highly expressed WRKY genes was also observed during early flag leaf development and may play roles in regulating developmental processes involved in leaf expansion and functionality (Additional file 2: Table S2).

### WRKY genes within co-expression modules

**Fig. 4:**
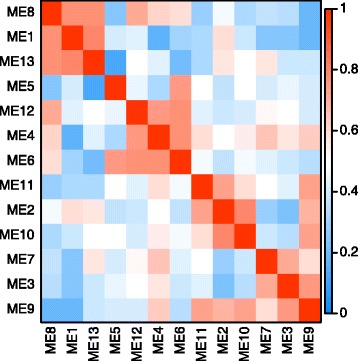
Module eigengene adjacency heatmap. Module-eigengenes (ME) in this analysis are defined as the first principal component of a coexpression module matrix. The heatmap shows the relatedness of the 13 co-expression modules (ME1-ME13) identified by WGCNA with red being highly related and blue being not related

Unsigned weighted gene co-expression network analysis (WGCNA) was utilized to place genes that were identified as differentially expressed over the course of flag leaf development (FDR < 0.05, log_2_ fold change > 1.5) into thirteen co-expression modules. Gene membership in the modules ranged from 5682 genes in module 1 to 21 genes in module 13. The expression profile of each module is summarized by a module eigengene, which is analogous to the first principal component of the module expression data. Comparison of the module eigengenes (Fig. 4, Additional file 3: Figure S1, Additional file 4: Figure S2) revealed four related module sets that displayed similar expression profiles across flag leaf development: (1) modules 1, 8, and 13; (2) modules 4, 5, 6, and 12; (3) modules 2, 10, and 11; (4) modules 3, 7, and 9. Key expression characteristics within each module set included high expression at (a) anthesis for set 1, (b) during seed development (seed set and mature seed) for set 2, (c) at heading for set 3, and (d) at the onset of senescence for set 4.

**Fig. 5:**
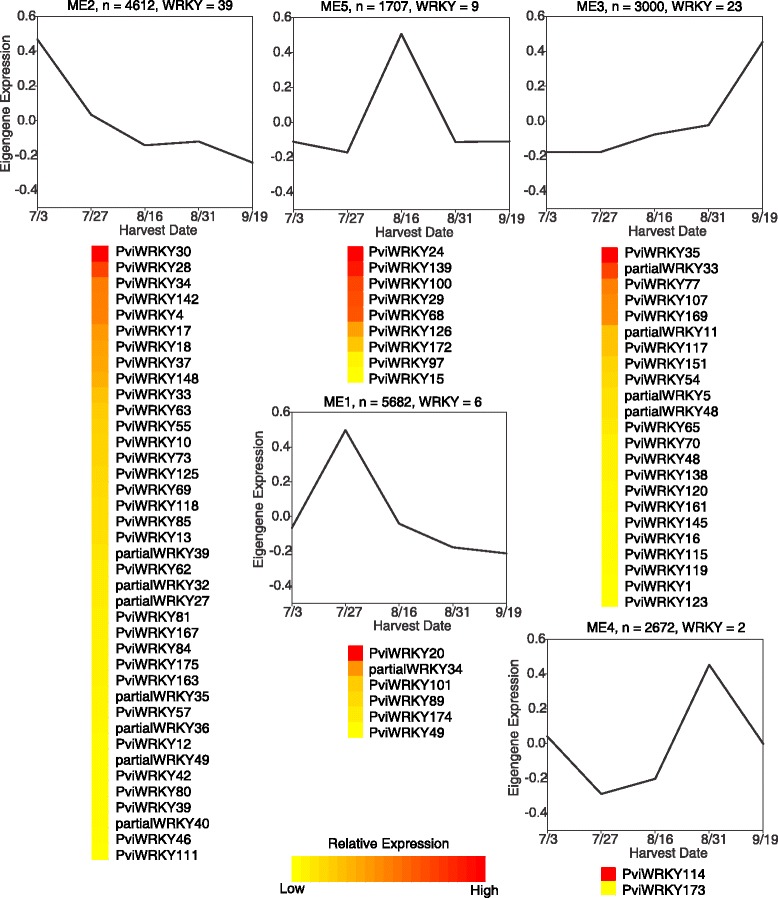
Select WRKY containing co-expression modules. Module eigengene expression profiles across the time series are shown, along with relative expression levels of individual WRKY genes within each select co-expression module. Red indicates high relative expression and yellow indicates low relative expression

A closer inspection of WRKY genes within the context of co-expression modules indicated that 79 out of 85 WRKYs included in WGCNA were assigned to five co-expression modules (Fig. 5). Module 2, whose expression peaked at heading (7/3/12) when flag leaves were still expanding [27], contained 39 WRKYs. Module 1 was the largest co-expression module and contained 5682 genes whose expression peaked at 7/27/12 coincident with anthesis and yet only contained six WRKYs. Nine WRKYs were found in module 5, whose expression levels peaked at seed set (8/16/12). This was a transition stage for flag leaves and plants as documented by the down regulation of genes linked to chlorophyll biosynthesis along with the appearance of a new sink tissue (seeds) respectively [27].

Two WRKYs were present in module 4, both with maximum expression at or around the time seeds attained physiological maturity [27]. Genes assigned to module 3, including 23 WRKYs (Fig. 5), had highest expression coinciding with senescence onset (9/19/12). These 23 WRKYs may be associated with senescence related processes.

### Senescence associated WRKY genes from switchgrass

**Fig. 6:**
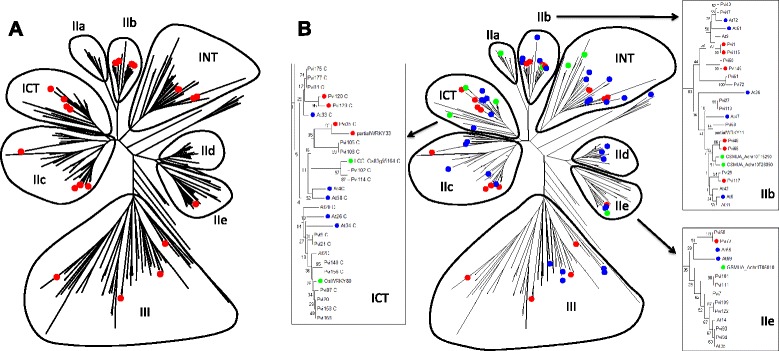
Senescence associated WRKY genes from switchgrass and other plants. **a** A combined Neighbor-Joining phylogenetic tree of all switchgrass and *A.thaliana* WRKY domain containing genes. The senescence-associated co-expression module 3 switchgrass genes are indicated in red. **b** A combined phylogenetic tree of all switchgrass and Arbabidopsis WRKY domains and several other senescence-inducible genes from other plants. The senescence-associated module 3 switchgrass genes are indicated in red, senescence-associated WRKY genes from *A. thaliana* (blue), switchgrass (red) and other plants (green) are shown with inset close ups of clusters of senescence associated WRKY genes. The other plants include rice, banana, and *Medicago truncatula*. Higher plants groups of WRKY genes (I-III) are shown. The evolutionary history was inferred using the Neighbor-Joining method. The optimal tree with the sum of branch length = 29.93 is shown. The tree is drawn to scale, with branch lengths in the same units as those of the evolutionary distances used to infer the phylogenetic tree

Figure 6a shows a combined phylogentic tree of the WRKY gene families from *A.thaliana* and switchgrass. As an example, the senescence-associated co-expression module 3 switchgrass genes are indicated in red. The WRKY genes from module 3 had no representatives from Groups IIa and only a single gene from Group IId and IIe. To verify if these patterns of WRKY associations were common to other plants as well, a number of senescence associated WRKY genes from rice, banana (*Musa acuminata*), and *Medicago truncatula* taken from the Leaf Senescence Database 2.0 (http://www.eplantsenescence.org/) were used for further analysis (Fig. 6b). Notably there are clusters of closely-related senescence associated WRKY genes in Group I, IIb, and IIc. Evidence supporting the possible involvement of these WRKY genes in regulating senescence comes from various data including expression analyses, mutants, and overexpression/knock down lines in several systems [11]. It is also possible that other WRKYs (for example module 5) are associated with senescence.

It is clear from Fig. 6b that there are local hot spots in the phylogeny where closely-related WRKY genes from multiple species are associated with senescence. This observation is particularly apparent for WRKYs belonging to Group I, IIb, and IIe. One hot spot in Group IIb contains three switchgrass WRKY genes (*PviWRKY48*, *PviWRKY65*, and *PviWRKY117*) together with two banana genes and two *A. thaliana* WRKY genes including the well-characterized regulator of senescence *AtWRKY6* [18, 19]. This suggests that *AtWRKY6*-like genes may regulate aspects of the senescence process across flowering plants, particularly as Fig. 6b shows representatives from both monocots and dicots. Group IIe is completely devoid of senescence-associated genes except for four very similar genes (*AtWRKY65*, *AtWRKY69*, *PviWRKY77*, and the banana gene *Achr1T05010*). This suggests that hot spots of senescence-associated genes are localized in the phylogenetic tree. However, it also suggests that possible function cannot be predicted from phylogenetic position alone as there are many instances such as *PviWRKY77* and *PviWRKY58* where apparent paralogs do not share the same association with senescence (Fig. 6b).

Taken together, the expression and phylogenetic studies have identified 23 switchgrass WRKY genes that show significantly enhanced mRNA levels during senescence of flag leaves under field conditions. Of these 23 genes, several are similar to senescence associated WRKY genes from other species and may represent conserved nodes in senescence signaling. Therefore, these genes represent potential targets for increasing biomass yields in switchgrass (and other flowering plants) by delaying senescence.

### Network visualizations across five different developmental states

**Fig. 7:**
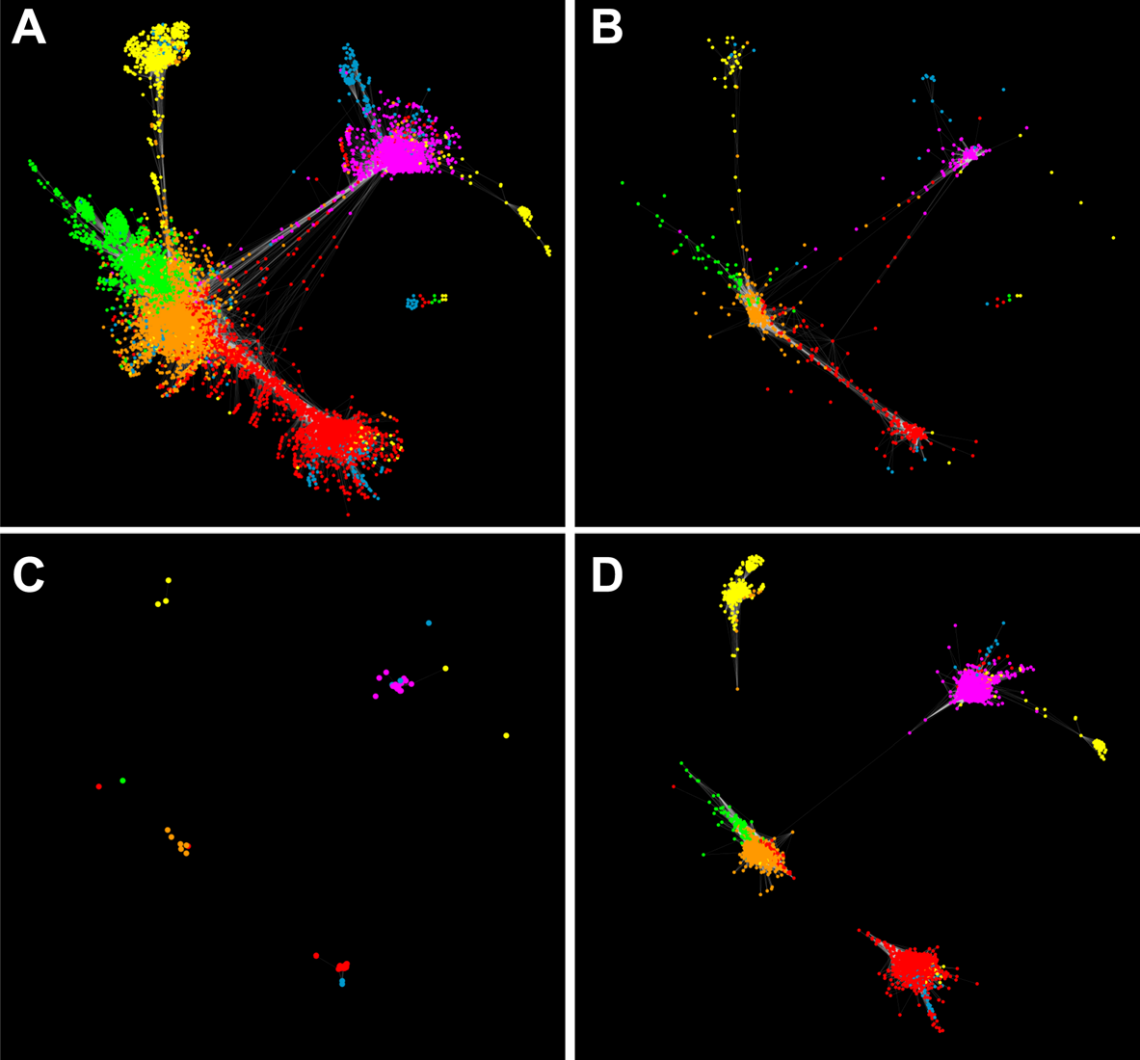
Edge-weighted network visualizations. **a** Each node represents an individual gene 13,405) and edges (428,378) connecting nodes are weighted by the topological overlap measure (TOM) as calculated by WGCNA. Nodes are colored based on the time point where each gene showed peak expression. Red = 7/3/12, Orange = 7/27/12, Yellow = 8/16/12, Green = 8/31/21, and Magenta = 9/19/12. **b** Only genes identified as transcript factors are shown. **c** Only WRKY transcription factors are shown. **d** Only WRKY genes and genes directly connected to WRKYs by an edge are shown. These are the genes with the highest degree of co-expression with the differentially expressed WRKYs

To better understand and identify co-expression clusters of genes regulated by the WRKYs, transcriptional networks were visualized. A transcription factor centered selection of 13,405 genes from the flag leaf expression data set [27] was used for these network analyses. These networks are shown in Fig. 7. The overall network patterns indicate that dynamic restructuring of the flag leaf transcriptomes was associated with key developmental events occurring both in the flag leaves as well as in the plant.

At the first harvest date, flag leaves were still expanding and had not yet transitioned into source leaves [27]. Genes with high expression during this time point (red; Fig. 7a) are linked to a cluster of genes associated with anthesis through a smaller network of genes (orange). At anthesis flag leaves had transitioned into source leaves [27], and the cluster of genes observed at this leaf developmental stage (orange; Fig. 7a) shares more connections to all the other network clusters (Fig. 7a). However, genes overexpressed in flag leaves at around the time of seed set (yellow; 8/16/12) were well separated from the central network hub, possibly because it was in response to an abiotic or biotic stress experienced by these plant/flag leaves around the collection date. Flag leaf gene networks when seeds were at the hard-seed stage (green; 8/31/12) were more closely aligned to the central network observed at anthesis (Fig. 7a) suggesting that transcriptional networks had possibly recalibrated after a stress-event observed the earlier stage. Genes with peak expression during senescence onset (magenta; 9/19/12) were distantly associated with the anthesis hub, although there were a few connections to the networks originating at around the time of seed set.

The 901 transcription factors forming the backbone of this entire network are highlighted (Fig. 7b) along with just WRKY transcription factors (Fig. 7c). The association patterns for the WRKYs are consistent with the involvement of specific WRKYs with specific stages of switchgrass flag leaf development. Interestingly, two WRKYs (yellow circles; Fig. 7c), namely *PviWRKY29* and *PviWRKY97* were more closely aligned to the WRKYs upregulated at the time of senescence onset (magenta circles; Fig. 7c). Genes directly connected to WRKYs by high topological overlap measure (TOM) value are depicted in Fig. 7d. Many of these genes could serve as direct targets for each respective WRKY.

To further investigate the relationships between WRKY expression patterns and cellular processes associated with flag leaf development, specific gene sets arising from the network analysis were performed to (a) evaluate the types and numbers of genes associated with senescence-related WRKYs and (b) searched for *cis*-acting elements in the available promoter sequences to identify putative W boxes that could serve as direct targets for WRKY transcription factors.

As described in Fig. 7c, although *PviWRKY29* and *PviWRKY97* were part of the genes overexpressed at the time of seed set, they were more closely aligned to the gene networks occurring at the time of senescence. *PviWRKY29* is found in the Group IIb senescence-associated hot spot and is a potential paralog of *PviWRKY117. PviWRKY29* and *PviWRKY117* are also the two apparent switchgrass orthologs of the well-known senescence regulator *AtWRKY6* (Fig. 6b) [18, 19]. PviWRKY97 is a Group I protein similar to the senescence-related WRKY protein encoded by the *OsWRKY80* gene (Fig. 6b).

There were 33 genes found with direct connections to *PviWRKY29* and *PviWRKY97*. Many of these genes encoded proteins destined to the cell-wall regions or were related to phosphate metabolism. Others appeared to be functioning in regulation/signal transduction. Of these genes directly linked to *PviWRKY29* and *PviWRKY97*, 23 had sufficient 5' upstream (putative promoter) sequence available. Nineteen of these 23 genes (Table 1) had one or more putative W boxes in these upstream sequences, suggesting they could be directly regulated by *PviWRKY29* and/or *PviWRKY97*. The genes where promoter regions are not currently available may also contain one or more W boxes, and the final number of genes that may be regulated by WRKYs could be higher.

**Table 1:** Potential W box containing genes in module 5 that were directly connected to *PviWRKY29* and *PviWRKY97* through co-expression analysis. Switchgrass gene ID, number of putative W boxes, the highest blast match to *A.thaliana* and functional description are given

Gene ID	No. W Box	Best match	Function description
Pavir.J02056	1	AT2G26190	calmodulin-binding family protein
Pavir.Da01490	2	AT5G47750	D6 protein kinase like 2
Pavir.J40688	1	AT4G39150	DNAJ heat shock N-terminal domain protein
Pavir.J24695	1	AT1G78060	Glycosyl hydrolase family protein
Pavir.Bb00101	2	AT4G05190	kinesin 5
Pavir.J13678	3	AT3G09220	laccase 7
Pavir.Cb01890	3	AT5G49760	Leucine-rich repeat protein kinase family
Pavir.Fa01734	3	AT1G56130	Leucine-rich repeat transmembrane kinase
Pavir.Fb01478	3	AT1G78130	Major facilitator superfamily protein
Pavir.J34655	1	AT2G37770	NAD(P)-linked oxidoreductase superfamily
Pavir.J38308	1	AT2G37770	NAD(P)-linked oxidoreductase superfamily
Pavir.Eb02929	1	AT5G11670	NADP-malic enzyme 2
Pavir.Ib03917	4	AT5G08030	PLC-like phosphodiesterases superfamily
Pavir.Ab01298	3	AT3G02040	senescence-related gene 3
Pavir.Da00281	3	AT2G20850	STRUBBELIG-receptor family 1
Pavir.Ba02108	2	AT2G01770	vacuolar iron transporter 1
Pavir.Cb01458	1	N/A	N/A
Pavir.J21234	1	N/A	N/A
Pavir.J09086	2	N/A	N/A

### *In-Silico* Promoter Analysis of Senescence-Related WRKY Genes

To determine potential environmental triggers that stimulate the expression of module 3 WRKY genes, the putative promoter region 1000 bp upstream of the start codon was scanned for *cis-*regulatory elements. All 17 senescence related WRKY genes with a putative promoter region from module 3, had a putative W box (TTGACY), suggesting auto and cross-regulation of WRKY genes. Additionally, putative ABA-responsive elements were present in multiple copies in the promoters of the majority of these module 3 WRKY genes, supporting their roles in activating cascades of genes involved in senescence. Common ABA-responsive motifs detected in the promoter regions of these WRKY genes included ACGTG, MACGYGB, ACGT, CCACGTGG, and ACGTSSSC. These sequences, if functional, would represent potential binding sites for several classes of transcription factors including bZIP and bHLHs. In addition to their proposed roles in triggering senescence, these WRKY genes also likely have roles in responding to abiotic stressors that serve as common triggers of senescence because motifs involved in drought responsiveness (DRE-1 and DRE-2), cold responsiveness (LTRE domains), phosphate starvation (P Starv), and sulfur responsiveness (Sulfur RE) were frequently detected in the promoter regions.

**Fig. 8:**
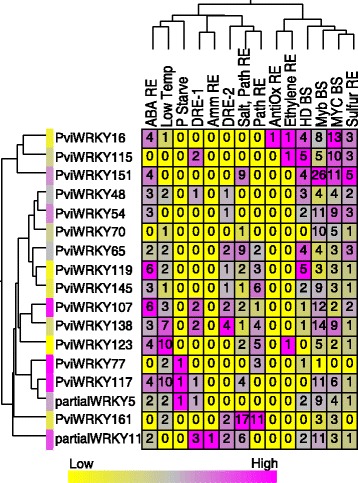
Promoter features of module 3 WRKYs. Seventeen of the 23 WRKYs in Module 3 had putative promoter regions which were further analyzed for the presence of additional motifs. Fourteen motifs (see methods for more details) were detected and the number of occurrences of each is shown along with the relative expression level of each WRKY (magenta being high, yellow being low). ABRE = Abscisic acid response element, Low Temp = Low temperature responsiveness, P Starve = Phosphate starvation response element, DRE-1 = Dehydration response element, Amm RE = Ammonium response element, DRE-2 = Dehydration responsive element, Salt, Path RE = Salt and Pathogen responsiveness element, Path RE = Pathogen responsiveness element, AntiOx RE = Antioxidant response element, Ethylene RE = Ethylene response element, HD BS = Homeodomain transcription factor binding site, Myb BS = Myb transcription factor binding site, MYC BS = MYC binding site, Sulfur RE = Sulfur responsive element

Further, the promoters of these WRKY genes contained putative regulatory elements for pathogen responsiveness (GT1GMSCAM4 and GCC box). These WRKY genes could represent a convergence point between the pathogen response and senescence pathways in switchgrass. WRKY transcription factors are known to play roles in both biotic and abiotic stress responses [11, 54, 55] and therefore the presence of putative W boxes in several WRKY gene promoters (Fig. 8) suggests that many of the senescence-associated WRKY genes represent common nodes between senescence and stress signaling pathways.

Our results present some evidence to support these common nodes between senescence and stress signaling pathways. Figure 7c shows that several WRKY genes from 8/16/12 (yellow) are more closely aligned to the gene networks occurring at the time of senescence (magenta). The yellow genes in Fig. 7 had been subjected to stress and this suggests that the yellow genes that map more closely with the senescence-associated genes are responsive to both stress and senescence. This includes *PviWRKY29* an apparent switchgrass ortholog of the well-characterized senescence regulator *AtWRKY6. AtWRKY6* is up-regulated by both pathogen attack and senescence and the results with *PviWRKY29* are therefore what would have been predicted for *AtWRKY6*.

## Discussion

### The WRKY gene family in switchgrass

WRKY transcription factors were identified in the whole-genome sequence of the tetraploid switchgrass clone AP13 (Version 1.1). Alamo, the cultivar from which AP13 originated, is extensively distributed throughout switchgrass breeding programs and is a heterozygous tetraploid with two sub-genomes designated A and B [56]. An identified total of 240 WRKY genes in tetraploid switchgrass is consistent with the total number of genes in rice, the most extensively characterized monocot species. Diploid rice contains approximately 125 WRKY genes [57] which is about half the number identified in switchgrass. Examination of the switchgrass WRKY phylogenetic tree depicted in Fig. 7 shows clearly that the majority of the genes are present in pairs that presumably represent A and B genes. Over the entire phylogenetic tree, about two thirds of senescence-associated switchgrass WRKY genes are present as A/B pairs, suggesting extensive conservation of function between similar genes in the A and B sub-genomes. Many of these pairs of genes also show similar senescence-associated expression characteristics (for example *PviWRKY1/PviWRKY115*, *PviWRKY120/PviWRKY123, PviWRKY35/PartialWRKY33* and *PviWRKY48/PviWRKY65*), although there are exceptions to this observation (*PviWRKY58/PviWRKY77, PviWRKY50/PviWRKY145,* and *PviWRKY29/PviWRKY117*).

Switchgrass contains both a Group RW6 resistance protein-WRKY (PviWRKY174) and a Group RW5 protein (PviWRKY178) [32]. R protein-WRKY genes appear to have evolved recently in flowering plants and each class appears to be restricted to specific flowering plant lineages [32], which suggests that RW5 and RW6 genes may have been present in the last common ancestor of switchgrass, sorghum and rice. *Brachypodium distachyon* appears to completely lack RW genes [29].

### Co-expression modules during leaf senescence

The role of WRKYs in a number of different plant developmental processes, especially in defense and senescence, is well established [11]. Senescence is a complex process and is influenced by a number of events both internal and external to the leaf [58]. Not unsurprisingly, many transcription factors, including WRKYs and NACs, can impact these processes. Often there is redundancy in the molecular events controlled by groups of transcription factors, suggesting that there is a dynamic balance in the interactions between these transcriptional regulators with each other and with the entire transcriptional machinery. More often than not, the specific functions of all the individual WRKYs sharing similar expression profiles are not known. However, high throughput expression analyses can provide some measure of understanding of these interactions.

The transcriptional datasets used in the current study were collected from switchgrass flag leaves at five different plant developmental stages from field grown plants [27]. Using NMDS, it was first established that WRKY gene expression within this dataset was consistent between replicates, indicating that flag leaves collected at each harvest dates were similar physiologically. In addition, NMDS analysis also established that the WRKYs were well differentiated at each harvest date, indicating that specific WRKYs were associated with cellular metabolism in flag leaves at different developmental stages. These differences were confirmed by the hierarchical clustering of the DE-WRKYs, which showed that specific WRKYs were up/down regulated in a manner that followed previously described changes in leaf physiology [27].

Through analysis of co-expression modules, it became possible to link changes in flag leaf transcription to specific physiological stages of flag leaf development. Of significance was the discovery of modules that were associated with leaf expansion (module 2), mature leaves (modules 1 and 5), pre-senescence (module 4) and senescence onset (module 3). Most WRKY genes were associated with early and late leaf development stages, consistent with their deduced roles in other species [11, 29, 59].

Module 4 which contained genes that had peak expression levels just before the onset of senescence only contained two WRKYs (*PviWRKY173 and PviWRKY114*). Although *PviWRKY173* was not highly expressed at this time point, *PviWRKY114* was. A closer inspection of *PviWRKY114* (Pavir.J00850) indicated that it has an unusual structure with an N-terminal DUF domain protein (domain of unknown function: DUF3598), and a C-terminal WRKY domain. This large fusion gene appears to be present in a few other plant genomes (for example, *Setaria italica* and rice). However, in many instances the two domains are located on independent genes. RNA-Seq mapping data indicated that reads were confined only to the N-terminal DUF3598 domain of Pavir.J00850 (data not shown), suggesting the WRKY domain is not expressed. These findings suggest that WRKY transcription factors may not be regulators of genes assigned to module 4.

### Senescence associated WRKY genes from switchgrass

A number of previous studies have shown the importance of WRKY transcription factors in regulating senescence [19, 60]. A recent study in cotton identified 3624 senescence-associated genes that showed differential expression during the process of senescence [61]. Of these genes, 519 encode transcription factors and the WRKY family had the most members associated with senescence (54) followed by bHLH (44), and C3H (42). Many members of the WRKY gene family were up-regulated early during the onset of senescence [61]. Unfortunately, only the raw Illumina reads from Lin et al. [61] are currently available and therefore it is not possible to include these data in our analysis. Nevertheless, it is clear that WRKY transcription factors are regulators of senescence [19, 60]. The 23 WRKY genes in module 3 are likely to include major regulators of senescence in switchgrass and represent excellent candidate genes for increasing switchgrass biomass by delaying senescence in the field.

*PviWRKY117* which was strongly associated with module 3 is an apparent switchgrass ortholog of *AtWRKY6* (Fig. 6b). *AtWRKY6* has been shown to positively regulate both senescence- and pathogen defense-associated genes [18]. One target gene of *AtWRKY6* is a receptor-like protein kinase (*FRK1*) whose expression is strongly induced during leaf senescence and is activated by AtWRKY6 binding to W boxes in the *FRK1* promoter. *PviWRKY117* had increased mRNA levels of over 13-fold during flag leaf development. The peak of its induction occurred at the last harvest date (9/19/12) when flag leaves were beginning to senesce. This expression profile is consistent with a role as a possible master regulator of senescence in switchgrass.

Inspection of the *PviWRKY117* demonstrated that its promoter contained known transcription factor binding sites, including a cluster of putative W boxes (underlined), a G box, and an S box (TTGACCCCATTGACC, CACGTGG, and AGCCACC). These are potential binding sites for WRKY, ERF, bZIP, and bHLH transcription factors. The presence of these sequences in the promoter of *PviWRKY117* suggests possible auto-regulation and previously *AtWRKY6* has been shown to suppress its own transcription [18].

### Network analyses provide robust evidence for the role of WRKYs during leaf development

Inferring functional relationships through co-expression and network analyses has already been a useful tool for the analysis of WRKY transcription factors [15–17]. Our network analyses provided a remarkable visual representation of the dynamic changes in flag leaf transcriptomes over time. Both the complexity of the connections as well as the apparent distinct molecular signatures at each major point in leaf development could be distinguished. Similar scenarios have been described for other monocot species [62–67].

Transcriptomic networks at early and late stages were especially well resolved and added to the overall interpretation of the changes in WRKY gene expression discussed above. As anticipated each developmental stage (harvest date) was linked to a number of different transcription factors, while WRKY-associated networks were particularly abundant during leaf expansion and the onset of senescence. Additionally, these analyses implied that specific WRKY transcription factors were strongly associated with specific flag leaf growth stages, and WRKY-controlled networks especially at an early and late stage of flag leaf development were generally independent. However, WRKYs present within module 5 associated with the time of seed set, presented an interesting profile possibly linking different cellular processes to the first molecular signatures for the onset of leaf senescence.

This specific module 5 subnetwork associated with the senescence cluster (see Fig. 7) contained only two WRKYs, namely *PviWRKY29* and *PviWRKY97*, which had direct connections to at least 19 genes that fell into two major categories: cell wall/defense and phosphate responsiveness. The role of WRKYs in plant defense is well established [11]. It is possible that the defense genes such as LRRs, a laccase, and a putative wall-bound xylosidase upregulated at this harvest date (8/16/12), (Pavir.J24695), were activated in response to an undetermined stress. Two other switchgrass genes, classified as NADP-linked oxidoreductase superfamily proteins, were found in this cluster. Related proteins have been indicated to have a direct role in the detoxification of stress-related accumulation of reactive carbonyls [68].

Recently, *A. thaliana AtWRKY45* [69] was shown to directly influence plant P levels through control of a phosphate transporter. A related WRKY, *A.thaliana AtWRKY42* exerted a greater influence on plant P status [70], and was suggested to impact plant P homeostasis. *PviWRKY29* is most similar to *A.thaliana AtWRKY6* and *AtWRKY42*, which both appear to impact plant P nutrition [70, 71], and *AtWRKY6* plays a role in leaf senescence [18], suggesting a potential link between early sensing of P status to the onset of senescence in switchgrass flag leaves. Pavir.Ab01298 (Table 1) is the switchgrass ortholog of the *A.thaliana senescence associated gene 3* (*SAG 3*), and encodes a phosphodiesterase. A similar gene plays a key role in maintaining plastid/cellular P homeostasis, especially under P starvation in *A.thaliana* [72].

Several other SAGs, including NADP-Malic enzyme 2, also associated with module 5 [27] providing evidence for a link between *PviWRKY29* and *PviWRKY97* and initiation of the senescence process. This expression network also contained a switchgrass ortholog of a vacuolar iron transporter gene. In rice, knockdowns of two leaf iron transporters led to increased iron translocation to the seeds [73]. However, switchgrass contains two sink tissues towards the end of a growing season, seeds and rhizomes [6]. Generally seeds are physiologically mature prior to completion of flag leaf senescence and rhizome dormancy [6, 27]. These observations raise the possibility that nutrient remobilization is staggered in switchgrass to meet the sink demands of different tissues.

Transcriptomic evidence for a causal relationship between WRKY expression and the onset of leaf senescence was seen at the last harvest date of flag-leaf sampling. Transcripts for 23 WRKYs were associated with the senescence-associated module 3, and the putative promoter regions for many of these WRKYs were populated with *cis*-elements known to confer response to both biotic and abiotic stresses. Several module 3 WRKYs were enriched for ABREs, DREs, low temperature, MYB, MYC, and sulfur-responsive elements, suggesting that module 3 WRKY gene expression was reflective of the internal and external environment during senescence onset. Further, these WRKYs were part of a network consisting of 3000 genes.

Among the SAGs within these networks were transcription factors, genes coding for enzymes in the chlorophyll degradation pathway, and several nutrient transporters with known roles during leaf senescence see Table 2) [27, 74–77]. Among these module 3 genes was a NAC transcription factor encoded by *Pavir.J16651*. This specific NAC (called PvNAC1) was demonstrated to impact leaf senescence in switchgrass [78], and is most similar to *AtNAC29*, which has been implicated in leaf senescence in *A. thaliana* [79].

**Table 2:** Potential Module 3 SAGs. SAGs were identified as described previously [27]. Other descriptions are as given for Table 1

Gene ID	Nearest At	At Description	Phase 4 RPKM
Pavir.Ba03899	AT1G26870	NAC domain containing protein 9	45
Pavir.Fb00689	AT2G33480	NAC domain containing protein 41	659
Pavir.Hb00869	AT3G04070	NAC domain containing protein 47	106
Pavir.J16651	AT1G69490	NAC domain containing protein 29	103
Pavir.Ca02775	AT3G12977	NAC domain containing protein	1270
Pavir.Ba01244	AT4G22920	non-yellowing 1	1280
Pavir.Gb00362	AT5G13800	pheophytinase	542
Pavir.Hb02058	AT5G13800	pheophytinase	387
Pavir.J04787	AT3G02040	senescence-related gene 3	367
Pavir.J37002	AT5G45890	senescence-associated gene 12	692
Pavir.Bb01489	AT5G45890	senescence-associated gene 12	1300
Pavir.Ab02441	AT5G24380	YELLOW STRIPE like 2	116
Pavir.Gb01191	AT5G53550	YELLOW STRIPE like 3	159
Pavir.Cb00745	AT2G03530	ureide permease 2	6913
Pavir.Ea02698	AT5G11670	NADP-malic enzyme 2	565
Pavir.J06980	AT3G45140	lipoxygenase 2	36
Pavir.J32181	AT2G42490	Copper amine oxidase family protein	54
Pavir.J16835	AT4G35090	catalase 2	163
Pavir.Fb00414	AT5G60360	aleurain-like protease	71
Pavir.Ia01427	AT4G36220	ferulic acid 5-hydroxylase 1	95

In addition to WRKY genes, Fischer-Kilbienski et al. have reported a protein containing the DUF548 domain (AtS40-3; AT4G18980) was targeted to the nucleus and regulated senescence via an *AtWRKY53*-dependent or independent route [80]. DUF548 is now recognized as a “senescence regulator" domain. The present version of the switchgrass genome contains 29 genes coding for proteins with the senescence regulator domain. Transcripts for 19 of these 29 genes were found in the flag leaf dataset, and only four of these genes were present in module 3 (Additional file 5: Figure S3). All four genes appeared to belong to group 1 proteins [80] (data not shown) indicating their importance to switchgrass flag leaf senescence. AtWRKY53 is a group III protein and of the large number of similar switchgrass genes, only *PviWRKY119* appears to be associated with senescence (Additional file 2: Table S2). However, it is possible that WRKYs could be part of the regulatory cascade influenced by the four switchgrass proteins containing the senescence regulator domain (Additional file 5: Figure S3).

Overall our analyses have classified all of the available full-length and partial switchgrass WRKY genes into specific protein clades and have placed their expression profiles within a framework of flag leaf development. More notably, it was possible to identify expression networks and expression modules that serve to integrate WRKY gene expression with specific genes. Many of these genes have known functions during leaf senescence in other plants. These findings provide a good platform for future analysis of specific genes and their ability to serve as markers for the continued improvement of switchgrass as a biofuel crop.

### Availability of supporting data

The data used in this manuscript are available as part of the short-reads archive depository within the NCBI at http://www.ncbi.nlm.nih.gov/sra/SRX481052/.

## Additional files

Additional file 1: Table S1. The WRKY gene family in switchgrass. (XLSX 25 kb) Additional file 2: Table S2. WRKY genes in the pro-senescence and early season clusters. (XLSX 9 kb) Additional file 3: Figure S1. Module Eigengene Dendrogram of the 13 modules (ME1-ME13) identified by WGCNA. (PDF 21 kb) Additional file 4: Figure S2. Expression profile of Module Eigengenes. (PDF 31 kb) Additional file 5: Figure S3. Expression heatmap of switchgrass genes coding for proteins containing the “senescence- regulator” domain. (PDF 27 kb)
