# Supplementary figures and images for: The WRKY transcription factor family and senescence in switchgrass
Source: BMC Genomics. 2015 Nov 9;16:912. doi: 10.1186/s12864-015-2057-4 (PMC4640240; doi:10.1186/s12864-015-2057-4)

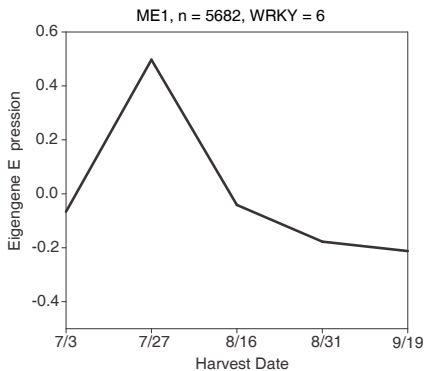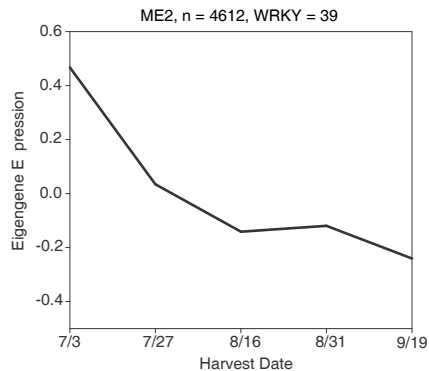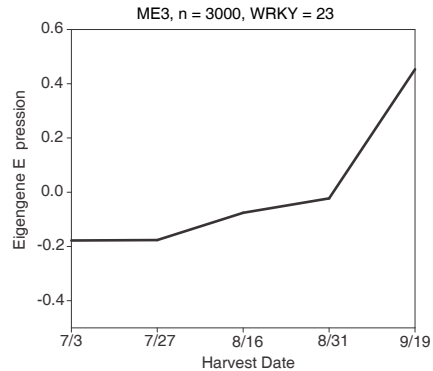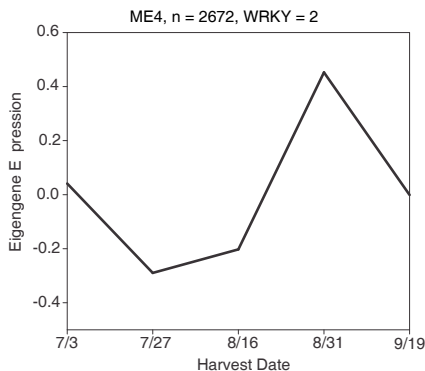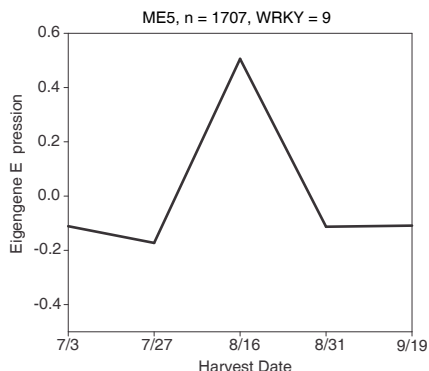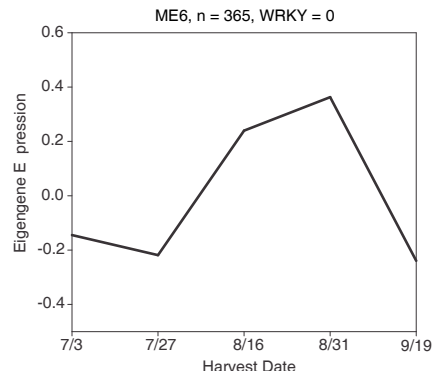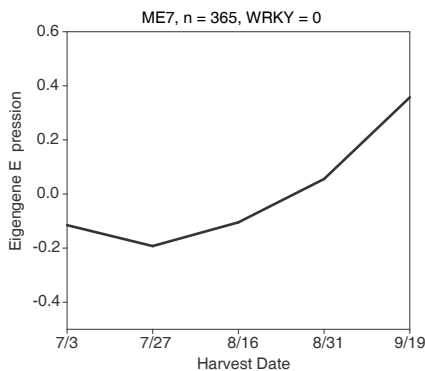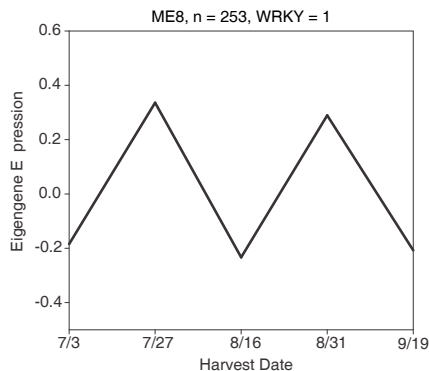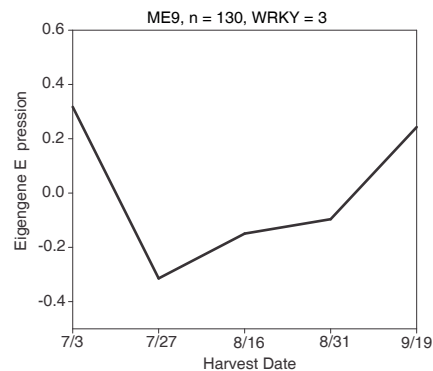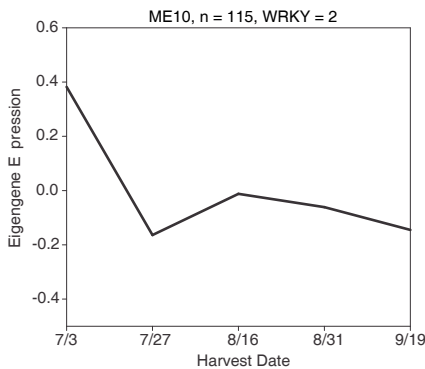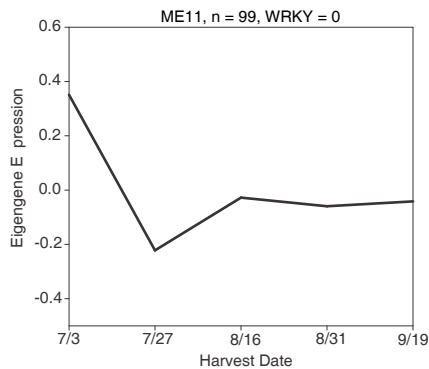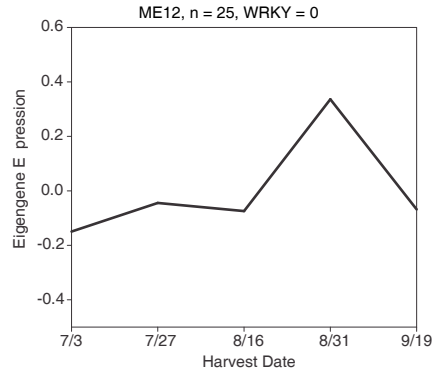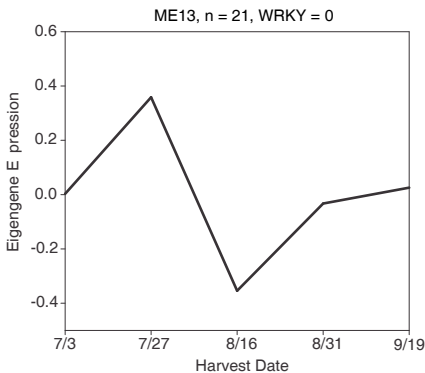

Supplement: Additional file 3: Figure S1. — Module Eigengene Dendrogram of the 13 modules (ME1-ME13) identified by WGCNA. (PDF 21 kb) [file 12864_2015_2057_MOESM3_ESM.pdf]

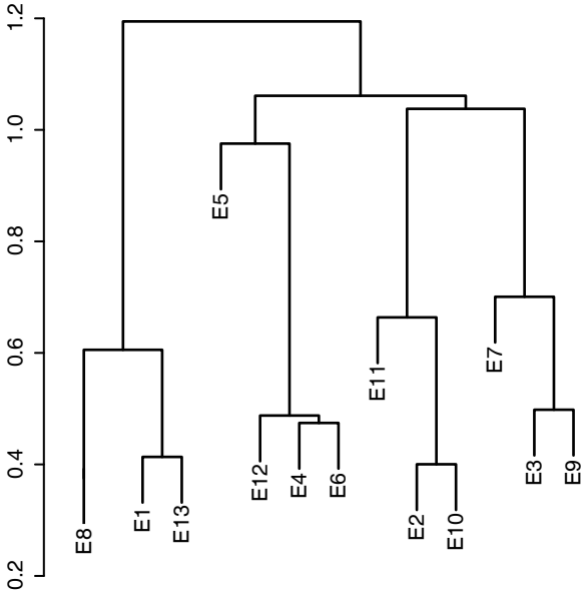

Supplement: Additional file 5: Figure S3. — Expression heatmap of switchgrass genes coding for proteins containing the “senescence- regulator” domain. (PDF 27 kb) [file 12864_2015_2057_MOESM5_ESM.pdf]
