# Supplementary material for: The WRKY transcription factor family and senescence in switchgrass
Source: BMC Genomics. 2015 Nov 9;16:912. doi: 10.1186/s12864-015-2057-4 (PMC4640240; doi:10.1186/s12864-015-2057-4)
Supplement: Additional file 4: Figure S2. — Expression profile of Module Eigengenes. (PDF 31 kb) [file 12864_2015_2057_MOESM4_ESM.pdf]

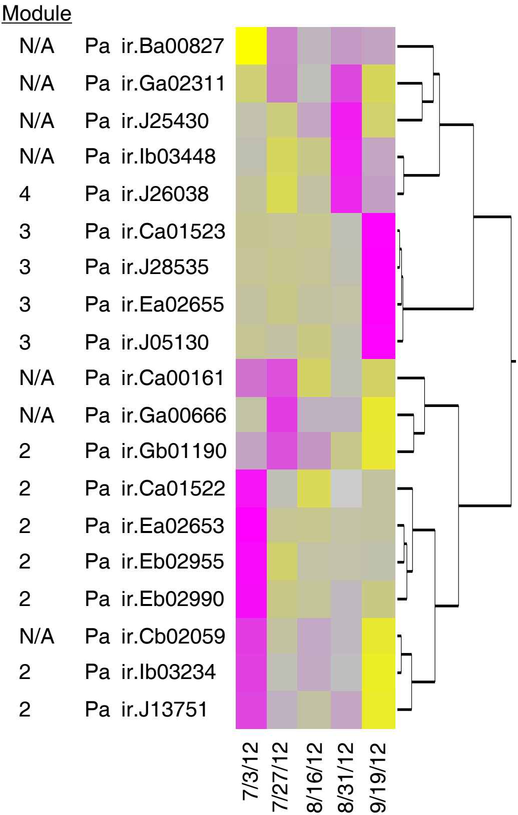
Module
N/A Pa ir.Ba00827
N/A Pa ir.Ga02311
N/A Pa ir.J25430
N/A Pa ir.Ib03448
4 Pa ir.J26038
3 Pa ir.Ca01523
3 Pa ir.J28535
3 Pa ir.Ea02655
3 Pa ir.J05130
N/A Pa ir.Ca00161
N/A Pa ir.Ga00666
2 Pa ir.Gb01190
2 Pa ir.Ca01522
2 Pa ir.Ea02653
2 Pa ir.Eb02955
2 Pa ir.Eb02990
N/A Pa ir.Cb02059
2 Pa ir.Ib03234
2 Pa ir.J13751
7/3/12
7/27/12
8/16/12
8/31/12
9/19/12
